# Screening of bacterial endophytes of larch against *Neofusicoccum laricinum* and validation of their safety

**DOI:** 10.1128/spectrum.04112-23

**Published:** 2024-06-24

**Authors:** Yuqian Liu, Shengjie Han, Liwen Song, Limei Li, Haifeng Wang, Min Pan, Jiajin Tan

**Affiliations:** 1 College of Forestry and Grassland, Collaborative Innovation Center of Modern Forestry in South China, Nanjing Forestry University, Nanjing, China; 2 Jilin Provincial Academy of Forestry Science, Changchun, China; 3 Forestry Bureau of Dunhua City, Jilin Province, Dunhua, China; USDA-ARS San Joaquin Valley Agricultural Sciences Center, Parlier, California, USA

**Keywords:** *Neofusicoccum flaricinum*, *Bacillus amyloliquefaciens*, biological control, antagonism, control effect, endophytes

## Abstract

In order to provide a highly feasible research pathway for the control of larch shoot blight, healthy larch branches and leaves were collected from 13 sampling sites in 8 provinces in China. The antagonistic endophytic bacteria obtained from the screening were used to carry out disease control experiments in potted seedlings. The safety evaluation test was conducted on the antagonistic bacteria. Subsequently, the strains with better preventive effect and high safety were identified by morphological and molecular methods. A total of 391 strains of endophytic bacteria were isolated from healthy larch branches and leaves. Seventy-eight strains of larch endophytic bacteria with antagonistic effect were obtained by primary sieving. Ten strains of endophytic bacteria with obvious antagonism were further obtained by secondary sieving, and all of them had an inhibition rate of more than 57%. Among them, strains YN 2, JL 6, NMG 23, and JL 54 showed the highest inhibition rate of 63.16%–65.08%, which was significantly different from the other treatments. The results of the pot test showed that 14 days after inoculation with the pathogen, strains YN 2 and JL 54 were more effective in the control of larch shoot blight, with the preventive effects reaching 57.7% and 50.0%, respectively. Strains JL 6 and JL 54 were biologically safe in the safety evaluation test. Therefore, strain JL 54 was selected for identification. It was identified as *Bacillus amyloliquefaciens* through morphological observation, 16S rDNA sequence, *gyrB* gene sequence and 16S rDNA-*gyrB* tandem feature sequence analysis.

**IMPORTANCE:**

Larch shoot blight is a widely distributed, damaging, and rapidly spreading fungal disease of forest trees that poses a serious threat to larch plantations. Endophytic bacteria have biological effects on host plants against pests and diseases, and they have a growth-promoting effect on plants. In this paper, we investigated for the first time the biocontrol effect of endophytic bacteria on larch shoot blight by screening endophytic bacteria with the function of antagonizing dieback fungi. *Bacillus amyloliquefaciens* JL 54 has a better prospect of biocontrol against larch shoot blight, which lays the foundation for the application of this bacterium in the future.

## INTRODUCTION

*Larix* spp. have strong adaptability, cold resistance, light preference, and fast growth rate. *Larix* spp. wood has tough material, detailed structure, weak causticity, and high economic value. Due to these characteristics, *Larix* spp. are frequently used as the preferred tree species for fast-growing and high-yield artificial timber forests.

Larch shoot blight is considered one of the most serious forest diseases worldwide, posing a severe threat to larch plantations (1). It was first discovered in Hokkaido, Japan, in 1938 (2, 3). In 1950, the pathogen was identified as *Physalospora laricina* Sawada (4). Subsequently, in 1961, Yamamoto and Kazuo recombined it into *Guignardia laricina* (Sawada) W. Yamam. & Kaz. Itô (2, 5). Shang suggested that it should be taken from the genus *Guignardia* and recombined into *Botryosphaeria laricina* (Sawada) Y.Z. Shang, which basically established its taxonomic status (6). This proposal was corroborated by Japanese forest pathologist Kobayashi, whose publication in 1990 confirmed the validity of the name (7, 8), subsequently adopted by contemporary researchers. Recent advancements in classification systems, particularly the establishment of Dothideomycetes, Botryosphaeriales, have provided further insights into its taxonomic positioning. Notably, in 2021, Hattori and Nakashima suggested the strain be re-classified as *Neofusicoccum laricinum* (Sawada) Y. Hattori & C. Nakash, signaling a noteworthy development in the taxonomic understanding of this pathogen (1).

The distribution of this disease encompasses regions in China, Japan, North Korea, South Korea, Russia, and other geographical locations. Larch shoot blight represents a very important fungal disease prevalent in northern Chinese larch plantations, characterized by its highly destructive nature and rapid dissemination. It has been designated as an official quarantine object in 2013 (9) and a key invasive alien species for management in 2023 (10) by China, which underscores its substantial impact. Moreover, the pathogen has also received classification as a quarantine pest from the European Food Safety Authority and the European and Mediterranean Plant Protection Organization (11, 12).

Larch shoot blight mainly affects the tops of the current year’s new growth of 1- to 35-year-old larch plantations but also causes serious damage to young larch forests of 6–15 years old. The disease typically initiates from the main shoots of larch and progresses downward from the upper crown. Upon disease onset, the tender stems of new shoots exhibit fading; leaves wither and fall off; and new shoots bend and droop, leaving only a limited number of needles at the apex (13–15).

In recent years, the prevalence of larch shoot blight has been increasing significantly, causing severe damage to larch trees. Currently, the primary methods for preventing and treating the disease involve chemical control and implementing appropriate forest management measures. However, these methods are not particularly effective in preventing the disease. Moreover, chemical control can only control the spread of the disease in a short period of time. However, there is a growing emphasis on biological control. It utilizes organisms that are harmless or beneficial to plants to resist pathogens, and affect or inhibit the survival and spread of pathogens (16). The diverse modes of action of biopesticides can effectively mitigate the rapid emergence of resistance in harmful organisms (17, 18), reduce environmental pollution, and sustain ecosystem integrity. In 2009, Liu et al. screened and obtained three fungi with significant antagonistic effects on larch shoot blight pathogens, demonstrating substantial control efficacy when applied through forest spraying (19). Subsequently, there has been a limited amount of research on biological control of larch shoot blight. Notably, no studies have investigated the biocontrol of plant endophytic bacteria for larch shoot blight.

Endophytic bacteria in plants primarily play key roles in diseases resistance, promoting plant growth, and facilitating symbiotic nitrogen fixation. This concept was initially introduced by Kloepper et al. in 1922, who highlighted that plant endophytic bacteria not only coexist with the plant for extended periods but also do not cause substantial damage to their host plants (20). Endophytic bacteria have the capacity to enhance the microbial environment while suppressing pathogen proliferation in *Pinus sylvestris*, thus significantly contributing to sustainable disease management (21). Throughout their coexistence with host plants, endophytes and their metabolites can stimulate host plants growth and bolster the hosts’ resistance and resilience to diseases.

In both field and greenhouse experiments, researchers demonstrated that the endophytic bacteria *Bacillus subtilis*, isolated from wheat, exhibited a potent control effect of 41.34% on *Gaeumannomyces graminis* var. tritici through root inoculation test, with the field control efficacy reaching 34.78% (22). Additionally, in 2000, Yi et al. discovered that an endophytic bacterium isolated from rice not only inhibited the mycelium of rice blight but also effectively suppressed the sclerotia of the pathogen (23). Furthermore, in 2003, Wang and Xiao identified an endophytic bacterium in tobacco that not only hindered the growth of *Phytophthora nicotianae* but also impeded the germination and movement of zoospores (24). Subsequently, in 2021, Ma et al. isolated and screened strains from *Saposhnikovia divaricata* (Turcz.) Schischk, which exhibited enhanced antagonistic effects against six pathogenic bacteria of ginseng, including *Rhizoctonia solani*, *Botrytis cinerea*, and *Phytophthora capsica* (25). Finally, in 2022, Wang et al. isolated endophytic bacteria from healthy pepper tissues for controlling *Fusarium oxysporum* in chili peppers (26).

Studying and utilizing endophytic bacteria with biocontrol effect against larch shoot blight can inhibit the occurrence of the disease and suppress pathogen proliferation. Therefore, in this study, we aimed to isolate and screen endophytic bacteria exhibiting potent antagonistic effects and high safety profiles, with the objective of demonstrating strong resistance to larch shoot blight. These efforts are intended to establish a foundation for future prevention and control measures against larch shoot blight.

## RESULTS

### Isolation of endophytic bacteria from larch

A total of 391 endophytic bacteria were isolated from healthy larch branches and leaves collected from 13 collection sites in 8 provinces. The number of strains collected from each sample point is provided in Table 1.

**TABLE 1:** The separation condition of endophytic bacteria from larch

Sampling province	Sampling site	Number of strains	Strain no.
Yunnan Province	Xiaoliangshan Yakou, Zhaotong City	28	YN 1-28
Hunan Province	Nanyue Forest Park, Hengyang City	7	HN 1-7
	Daxiongshan National Forest Park, Loudi City	20	HN 8-27
Hebei Province	Saihanba Machinery Forest Farm, Chengde City	40	HB 1-40
Jilin Province	Liangshui Village, Tonghua City	23	JL 1-23
	Mudangang Forest Farm, Dunhua City	63	JL 24-86
Liaoning Province	Shenyang Agricultural University Botanical Garden, Shenyang City	58	LN 1-58
Heilongjiang Province	Jianshanzi, Shangzhi City	19	HLJ 1-19
Inner Mongolia Autonomous Region	Huanggangliang Forest Farm, Chifeng City	47	NMG 1-47
	Huamugou National Forest Park, Chifeng City	23	NMG 48-70
Shandong Province	Wanshougong Forest Farm, Linyi City	35	SD 1-35
	Zhongshan Park, Qingdao City	15	SD 36-50
	Taipingshan Central Park Botanical Garden, Qingdao City	13	SD 51-63

### Screening of endophytic antagonistic bacteria in larch

**Fig 1:**
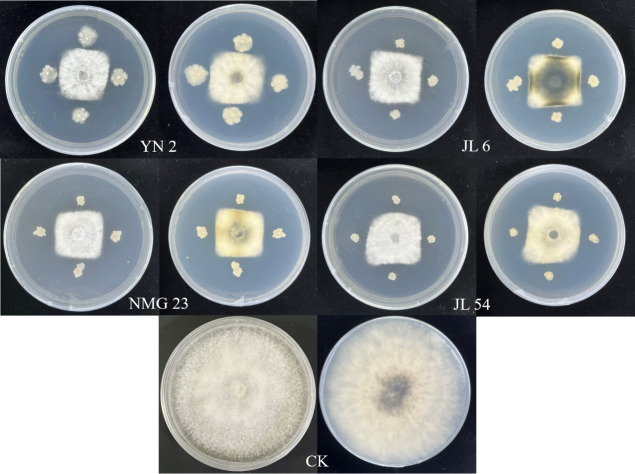
Effect of four antagonistic endophytic bacteria against *Neofusicoccum laricinum.*

Initial screening using the two-sided lineation confrontation culture method yielded 78 strains of endophytic bacteria exhibiting significant antagonistic effects. Further re-screening using the four-point confrontation culture method resulted in the selection of 10 strains with strong antagonistic effects. The inhibition rates of these strains exceeded 57% (Table 2). Based on the subsequent re-screening, four strains of bacteria YN 2, JL 6, NMG 23, and JL 54, demonstrating the most potent antagonistic effects, were chosen for subsequent pot seedling control experiments. The inhibition rates of these strains ranged from 63.16% to 65.08 %, with all rates surpassing 63% (Table 2; Fig. 1).

**TABLE 2:** Inhibition of *Neofusicoccum laricinum* by endophytic bacteria of larch obtained after re-screening^*a*^

Strain	Pathogen colony diameter (mm)	Inhibition rate (%）
YN 2	31.43 ± 1.20 c	65.08 ± 1.33 a
JL 6	31.87 ± 2.51 c	64.59 ± 2.79 a
NMG 23	31.99 ± 1.14 c	64.46 ± 1.27 a
JL 54	33.16 ± 2.67 bc	63.16 ± 2.97 ab
YN 10	35.67 ± 2.08 ab	60.12 ± 1.75 bc
SD 16	36.16 ± 1.04 ab	59.40 ± 2.78 bc
JL 1	36.80 ± 2.04 a	59.12 ± 2.26 bc
HN 3	37.78 ± 2.18 a	58.02 ± 2.42 c
HN 16	38.34 ± 0.63 a	57.19 ± 0.69 c
JL 4	38.52 ± 1.10 a	57.20 ± 1.23 c

^*a*^ The data in the table are the averages of three replicates ± standard deviation. Data with the same letters mean the differences are not significant in the 5% level (Duncan multiple range test).

### The control effect of antagonistic endophytic bacteria against *Neofusicoccum laricinum*

After 7 days of inoculation with *Neofusicoccum laricinum*, the control group inoculated with the pathogen began to exhibit disease symptoms, with yellowing needles observed. In contrast, the pine seedlings in the treatment group did not display any signs of development. By day 9 of inoculation, the symptoms in the control group became more apparent as the needles of the diseased seedlings started to dry up and fall off. The treatment group, on the other hand, showed indications of yellowing. After 14 days of inoculation, the incidence rate in the control group reached 100%, with a disease index of 72.22. Some needles of in the treatment group experienced needle drying and shedding, but to a lesser extent compared to the control group. The incidence rate in the treatment group was below 80%, and the disease index remained below 45. Notably, the treatment group inoculated with endophytic bacteria YN 2 exhibited the most effective control, with a rate of 57.7%. Endophytic bacteria JL 54 had a control effect of 50.0%, while JL 6 and NMG 23 exhibited control effects of 38.5% and 42.3%, respectively (Table 3). The growth of pine seedlings in the blank control group remained normal, with no observable changes in their needles. In the pot seedling test, both the YN 2 treatment group and the JL 54 treatment group demonstrated better control effects against larch shoot blight.

**TABLE 3:** Control effect of different antagonistic strain to larch shoot blight^*a*^

Treatment	Disease index	Incidence (%)	Control effect (%)
YN 2	30.56 ± 10.02 b	66.67	57.68
JL 6	44.44 ± 10.24 b	83.33	38.47
NMG 23	41.67 ± 5.69 b	83.33	42.30
JL 54	36.11 ± 13.21 b	83.33	50.00
CK1	72.22 ± 5.56 a	100	
CK2	0 c	0	

^*a*^ The data in the table are the averages of three replicates ± standard deviation. Data with the same letters mean the differences are not significant in the 5% level (Duncan multiple range test). YN 2 denotes inoculation with the antagonistic bacterium YN 2 and *Neofusicoccum laricinum*; JL 6 denotes inoculation with the antagonistic bacterium JL 6 and *Neofusicoccum laricinum*; NMG 23 denotes inoculation with the antagonistic bacterium NMG 23 and *Neofusicoccum laricinum*; JL 54 denotes inoculation with the antagonistic bacterium JL 54 and *Neofusicoccum laricinum*; CK1 denotes inoculation with *Neofusicoccum laricinum* only; CK2 denotes non-inoculation.

### Safety test results of four antagonistic bacteria

#### Tobacco inoculation

**Fig 2:**
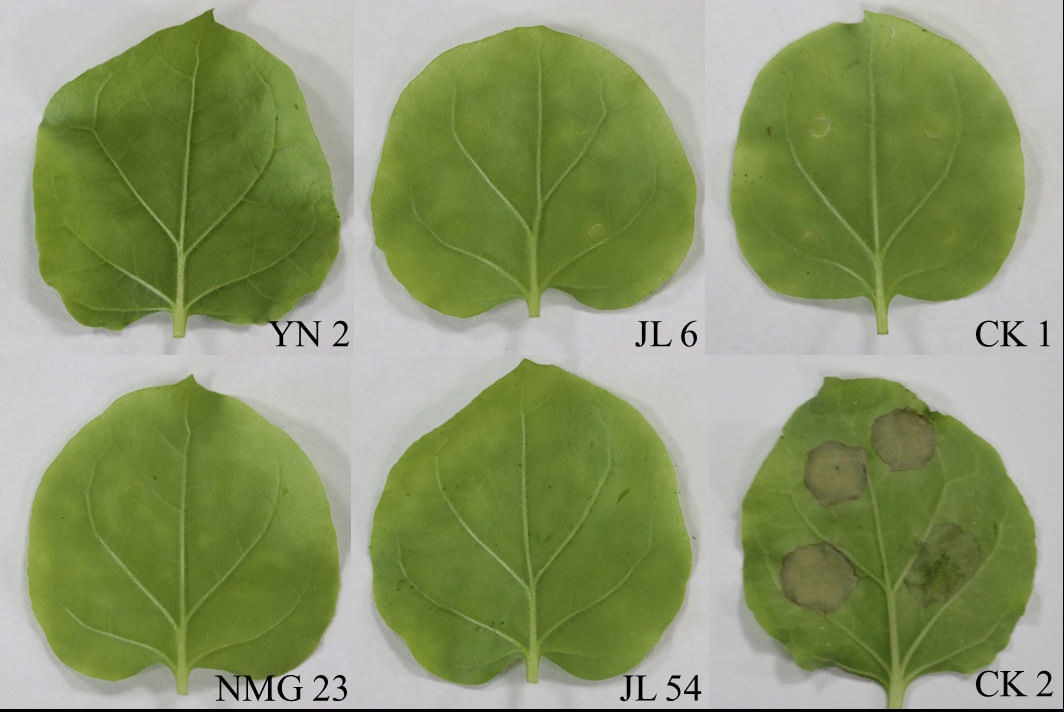
Pathogenicity of four strains of endophytic bacteria on tobacco leaf. CK 1 (blank control): inoculated with sterile water; CK 2 (negative control): inoculated with tobacco pathogen.

The results of tobacco inoculation with the antagonistic strains YN 2, JL 6, NMG 23, and JL 54 are presented in Fig. 2. The tobacco leaves inoculated with sterile water in the control group (CK1) exhibited only slight stains at the inoculation site without any disease symptoms. In contrast, the tobacco leaves inoculated with the pathogenic strains in the control group (CK2) displayed evident brown discoloration at the inoculation site, adversely affecting tobacco growth. Similarly, the tobacco leaves inoculated with the four antagonistic bacteria strains showed no lesions and were comparable to the blank control, with only water stains observed at the inoculation site. Furthermore, previous inoculation tests confirmed that these four antagonistic bacteria did not pose any toxicity to potted larch seedlings. These findings collectively indicate that the four antagonistic bacteria are non-pathogenic to tobacco plants.

#### Alfalfa inoculation

**Fig 3:**
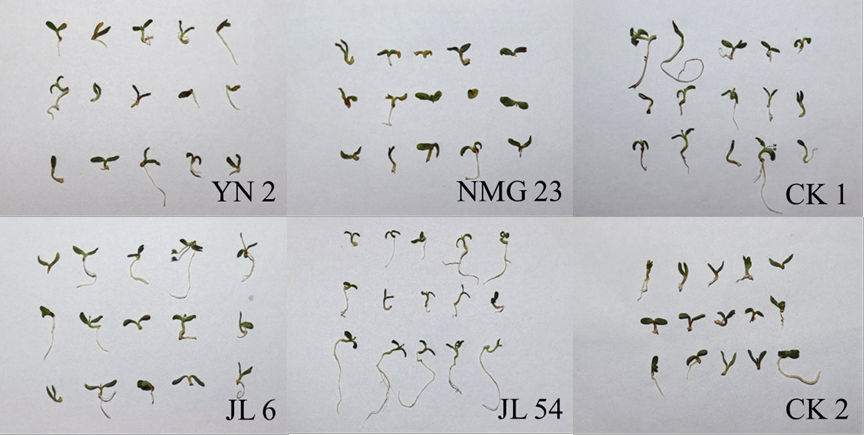
The growth status on alfalfa seedlings after 12 days of inoculation of four strains of endophytic bacteria. CK 1 (blank control): Inoculated with LB liquid medium; CK 2 (negative control): inoculated with pathogenic strain LMG 1222.

The results of alfalfa inoculation revealed that alfalfa seedlings inoculated with Luria-Bertani (LB) liquid medium did not exhibit any disease symptoms. In contrast, the seedlings inoculated with the pathogenic strain LMG 1222 (control strain with pathogenic activity on alfalfa) displayed yellowing and shortened roots, which hindered alfalfa growth. The seedlings inoculated with strains YN 2 and JL 6 showed no symptoms of disease. The seedlings inoculated with strain JL 54 grew better than the blank control (inoculated with LB liquid medium), indicating it has a potential growth-promoting effect on alfalfa. On the other hand, the seedlings inoculated with strain NMG 23 presented more severe symptoms of yellowing and necrosis than LMG 1222, significantly inhibiting the growth of alfalfa(Fig. 3). These findings indicate that the antagonistic strains YN 2, JL 6, and JL 54 are non-pathogenic to alfalfa and demonstrate high safety. However, the antagonistic strain NMG 23 exhibits strong pathogenicity toward alfalfa and low safety.

#### Drug sensitivity test

**Fig 4:**
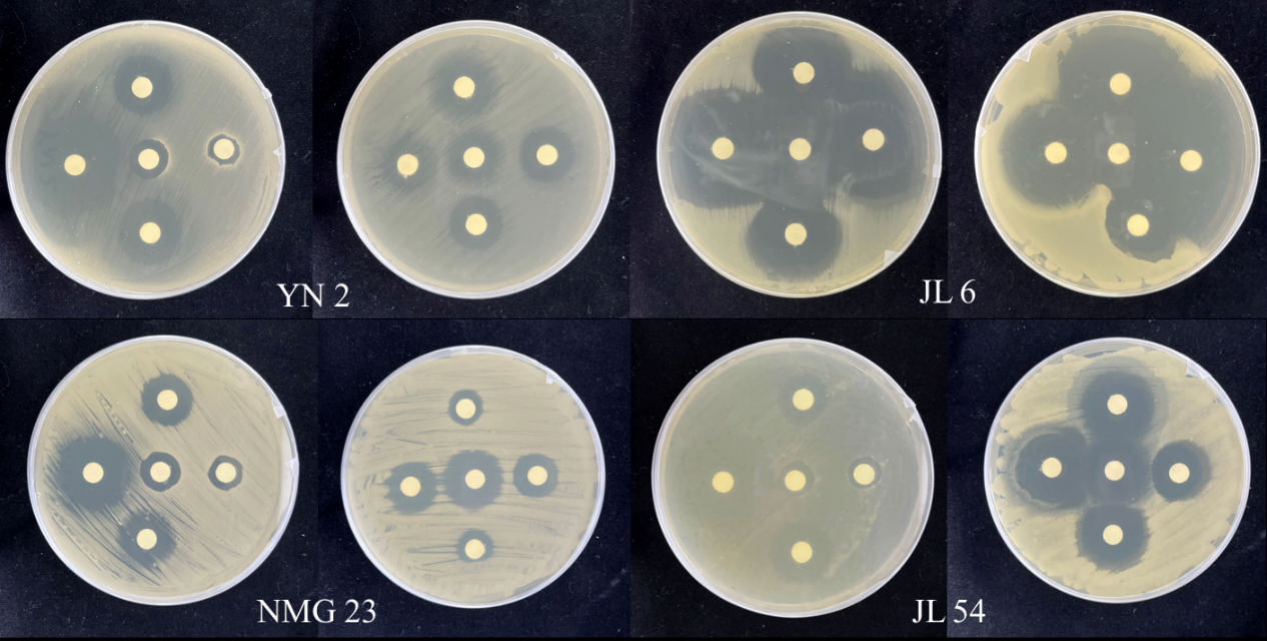
Drug sensitivity test of four strains of endophytic bacteria.

The results indicate that strain YN 2 demonstrated sensitivity to seven antimicrobial drugs, namely, cefazolin, amikacin, gentamicin, norfloxacin, ciprofloxacin, cotrimoxazole, and chloramphenicol. However, it exhibited resistance to two antimicrobial drugs: penicillin and ampicillin. Strain JL 6 displayed sensitivity to eight antimicrobial drugs, including ampicillin, cefazolin, amikacin, gentamicin, norfloxacin, ciprofloxacin, cotrimoxazole, and chloramphenicol, but showed resistance to penicillin and erythromycin. Strain NMG 23 showcased sensitivity to four antimicrobial drugs, namely, cefazolin, amikacin, gentamicin and cotrimoxazole, but showed resistance to penicillin, ampicillin, norfloxacin, and chloramphenicol. Similarly, strain JL 54 exhibited sensitivity to eight antimicrobial drugs, namely, cefazolin, amikacin, gentamicin, erythromycin, norfloxacin, ciprofloxacin, cotrimoxazole, and chloramphenicol, but showed resistance to penicillin and ampicillin (Table 4, Fig. 4).

**TABLE 4:** Drug sensitivity test results of four strains of endophytic bacteria^*a*^

Antibiotics	Paper drug content (μg/tablet)	Diameter of inhibition circle (mm) (sensitivity)			
		YN 2	JL 6	NMG 23	JL 54
Penicillin	10	10.11 ± 0.11 (R)	14.26 ± 0.27 (R)	11.17 ± 0.28 (R)	10.68 ± 0.30 (R)
Ampicillin	10	13.12 ± 0.51 (R)	18.32 ± 1.43 (S)	13.30 ± 0.40 (R)	11.80 ± 0.42 (R)
Cafamezin	30	48.87 ± 8.63 (S)	47.35 ± 1.21 (S)	23.07 ± 0.25 (S)	31.40 ± 1.04 (S)
Amikacin	30	29.46 ± 0.82 (S)	29.59 ± 0.83 (S)	17.03 ± 0.02 (S)	16.61 ± 0.36 (S)
Gentamicin	10	19.86 ± 1.06 (S)	23.84 ± 1.06 (S)	15.09 ± 0.21 (S)	19.92 ± 0.42 (S)
Erythromycin	15	21.61 ± 0.72 (I)	13.64 ± 0.96 (R)	20.08 ± 0.66 (I)	23.19 ± 1.29 (S)
Norfloxacin	10	16.39 ± 0.60 (S)	26.85 ± 1.03 (S)	11.55 ± 0.57 (R)	24.55 ± 1.24 (S)
Ciprofloxacin	5	25.69 ± 3.23 (S)	36.17 ± 1.09 (S)	17.74 ± 0.88 (I)	20.02 ± 0.28 (S)
Compound sulfamethoxazole	25	19.67 ± 1.67 (S)	24.95 ± 0.53 (S)	17.52 ± 0.50 (S)	26.37 ± 0.71 (S)
Chloroamphenicol	30	23.78 ± 0.63 (S)	27.38 ± 1.73 (S)	10.57 ± 0.63 (R)	25.25 ± 0.43 (S)

^*a*^ I, sensitive intermediary; R, resistant; S, sensitive.

According to the General Criteria for Quality and Safety Evaluation of Microbial Fertilizers (standard no. GB/T 41728–2022), all four strains demonstrated sensitivity to two or more antimicrobial agents, which is a requirement. Additionally, complying with the General Technical Guidelines for Biosafety of Microbial Fertilizers (standard no. NY/T 1109–2017), these strains must exhibit sensitivity to more than two antimicrobial agents.

#### Hemolysis test

**Fig 5:**
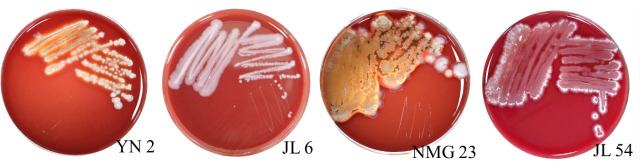
Hemolysis test of four strains of endophytic bacteria.

The hemolysis test results revealed that strains JL 6 and JL 54 did not exhibit any hemolysis around their colonies on the blood plate, and no hemolysis ring was formed. Conversely, a yellowish-green semi-transparent hemolysis ring was observed around the colonies of strain YN 2. This α-hemolysis phenomenon was attributed to methemoglobin, and the red blood cells within the hemolysis were not completely dissolved. In contrast, a distinct and fully transparent hemolytic ring measuring 2–4 mm in diameter was observed around the colonies of strain NMG 23, indicating a β-hemolytic phenomenon. This observation suggests that strain NMG 23 produces hemolysin capable of completely dissolving red blood cells during culture (Fig. 5).

The hemolytic activity of four strains of endophytic bacteria was determined by using defibrinated sheep blood erythrocytes. The hemolysis rate of strain NMG 23 was as high as 44.58% ± 0.0956%, significantly higher than other strains (Table 5). It was far exceeding the national standard requirements. The hemolysis rate of strain YN 2 was 15.90% ± 0.0124%. It also had a little hemolytic activity. The hemolysis rates of strains JL 6 and JL 54 were both lower than the national standard of 5%, which is considered biosafety. It can also be seen that the results of the two hemolytic activity judgment methods are consistent.

**TABLE 5:** The results of hemolysis rate of four strains of endophytic bacteria^*a*^

Treatment	Absorbance (OD_545_)	Hemolysis rate (%)
YN 2	0.1202 ± 0.0086 c	15.90 ± 0.0124 b
JL 6	0.0396 ± 0.0088 d	4.19 ± 0.0128 c
NMG 23	0.3178 ± 0.0659 b	44.58 ± 0.0956 a
JL 54	0.0261 ± 0.0055 d	2.24 ± 0.0079 c
CK1	0.0107 ± 0.0009 d	
CK2	0.6997 ± 0.0005 a	

^*a*^ CK 1 (negative control) denotes physiological saline; CK 2 (positive control) denotes distilled water.

#### Detection of toxin genes

The four antagonistic bacteria were found to possess the hemolytic enterotoxin gene, non-hemolytic enterotoxin gene, vomitoxin gene, enterotoxin *FM* gene, and cytotoxin *K*. Both hemolytic enterotoxin and non-hemolytic enterotoxin are complex proteins consisting of multiple subunits, while the vomitoxin enterotoxin *FM* gene and cytotoxin *K* are monomeric proteins.

**Fig 6:**
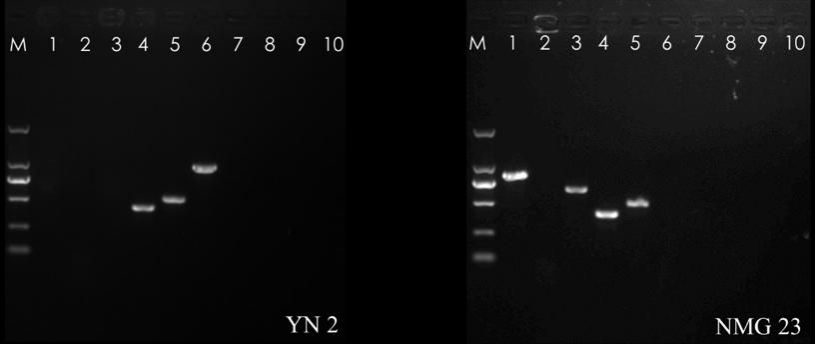
PCR amplification of the virulence genes of strains YN 2 and NMG 23. M: DL 2000 DNA marker; 1, *hblA*; 2, *hblB*; 3, *hblC*; 4, *hblD*; 5, *nheA*; 6, *nheB*; 7, *nheC*; 8, *ces*；9, *entFM*; 10, *cytK*.

However, neither strain JL 6 nor JL 54 exhibited the presence of the aforementioned virulence genes (Table 6). In contrast, strain YN 2 was found to have the hemolytic enterotoxin gene (*hblD*) and non-hemolytic enterotoxin genes (*nheA* and *nheB*). Additionally, strain NMG 23 displayed the presence of hemolytic enterotoxin genes (*hblA*, *hblC*, and *hblD*) and non-hemolytic enterotoxin gene (*nheA*) (Fig. 6), indicating that strain NMG 23 has the ability to secrete *Bacillus hemolyticus*.

**TABLE 6:** PCR amplification of the virulence genes of four strains of endophytic bacteria^*a*^

Toxin gene		YN 2	JL 6	NMG 23	JL 54
Hemolytic enterotoxin genes	*hblA*	-	-	+	-
	*hblB*	-	-	-	-
	*hblC*	-	-	+	-
	*hblD*	+	-	+	-
Non-hemolytic enterotoxin genes	*nheA*	+	-	+	-
	*nheB*	+	-	-	-
	*nheC*	-	-	-	-
Vomitoxin gene	*ces*	-	-	-	-
Enterotoxin *FM* gene	*entFM*	-	-	-	-
Cytotoxin *K*	*cytK*	-	-	-	-

^*a*^ “+” indicates that the strain contains the gene; “-” indicates that the strain does not contain the gene.

### Identification of significant antagonistic bacterium

#### Morphological characterization of strain

**Fig 7:**
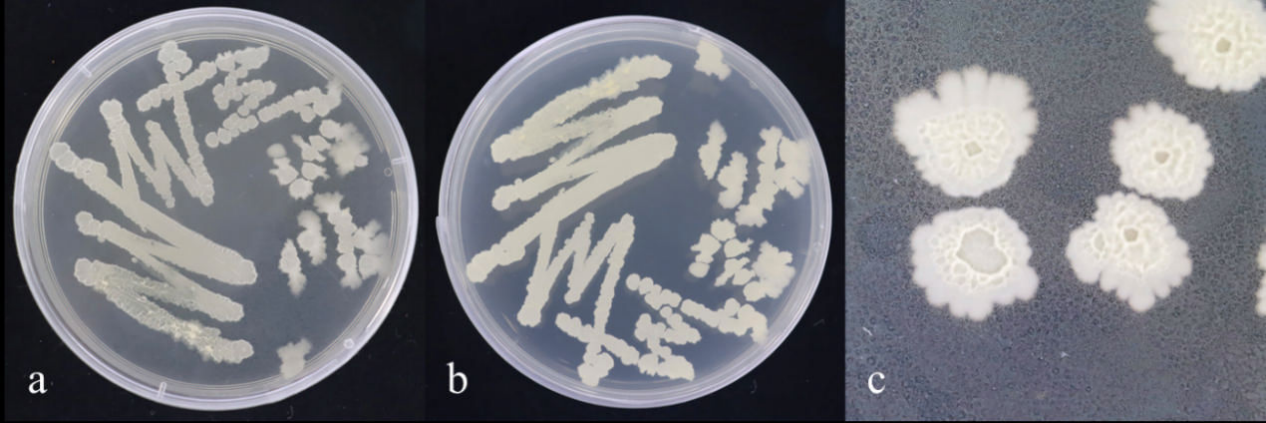
Morphological characteristics of strain JL 54 on LB medium. (a and b) Bacterial lawn morphology of strain JL 54 cultured on NA solid medium for 48 h. (c) Single-colony morphology of strain JL 54 cultured on NA solid medium for 7 days.

**Fig 8:**
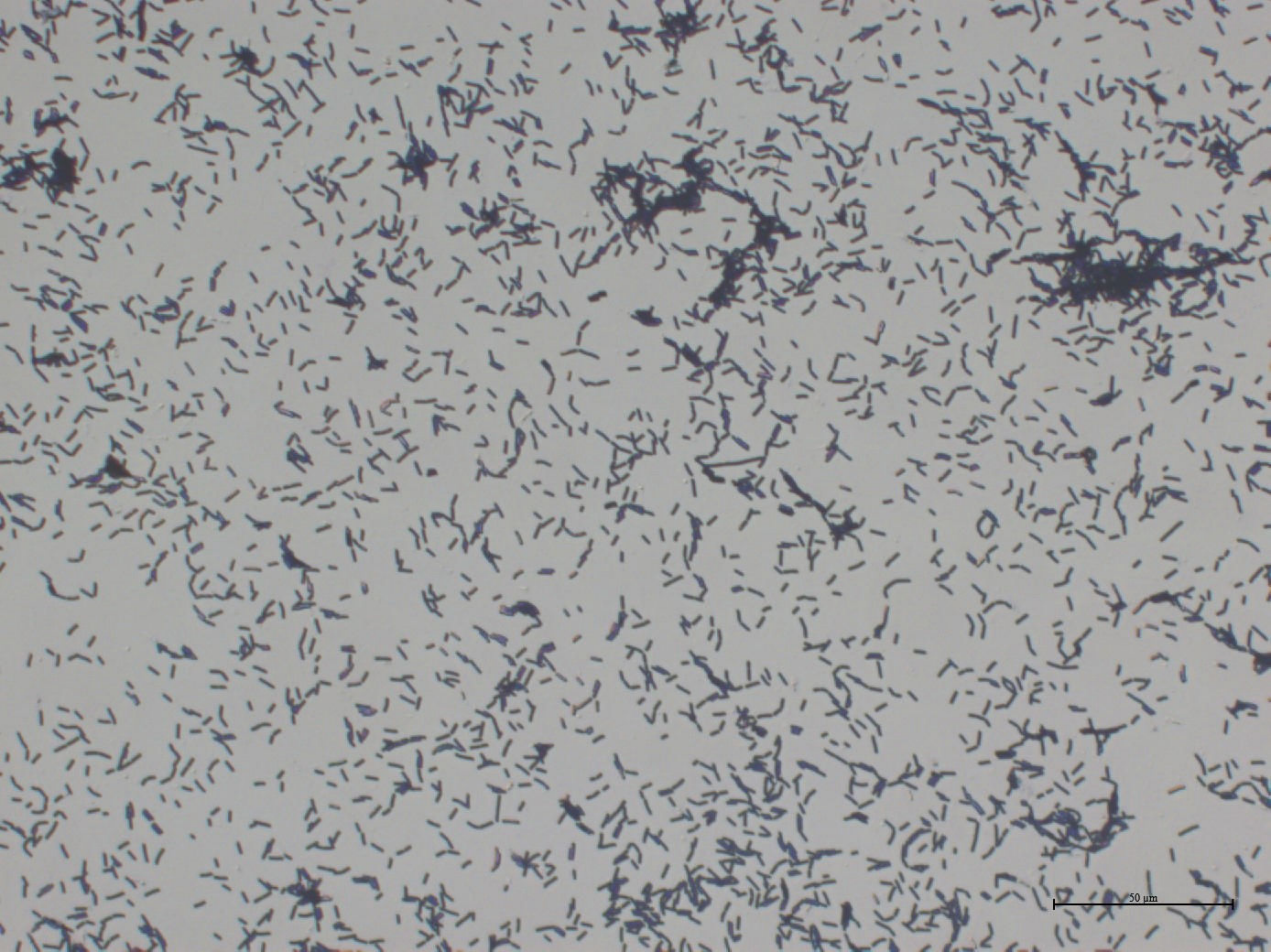
Gram staining results of strain JL 54.

After 48 h of cultivation on Nutrient Agar (NA) medium, strain JL 54 formed colonies that were slightly yellow and opaque, with rough surface and irregular edges. Following 7 days of cultivation on NA medium, the single colonies exhibited a milky white and opaque appearance, with a rough and wrinkled surface, wavy edges, and a colony diameter ranging from 2 to 6 mm (Fig. 7). Microscopic examination revealed rod-shaped cells that were Gram positive (Fig. 8).

#### 16S rDNA sequence analysis and phylogenetic tree construction

To identify the strain, PCR amplification was conducted using 16S rDNA specific primers with strain JL 54 as the template. The resulting amplification products were sent for sequencing at Sangon Biotech (Shanghai). Subsequently, DNA MAN software was utilized to assemble the sequencing data from the upstream and downstream primers. The obtained target sequence was found to be 1,455 bp long. The 16S rDNA sequence information of strain JL 54 is available from National Center for Biotechnology Information (NCBI) under GenBank accession no. PP690781.

**Fig 9:**
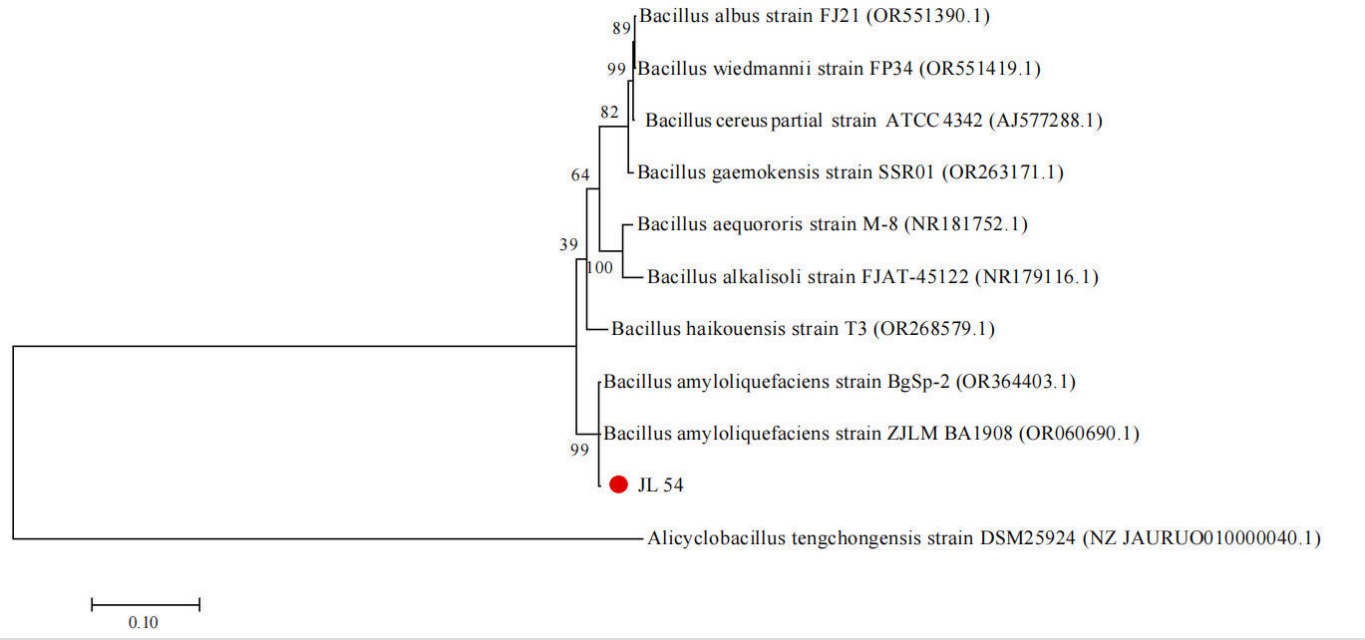
Phylogenetic tree of strain JL 54 based on BLAST results of the 16S rDNA sequence.

We conducted a Basic Local Alignment Search Tool (BLAST) similarity alignment with known sequences on NCBI. The results showed that the 16S rDNA sequence of strain JL 54 had the highest homology with *Bacillus amyloliquefaciens*, with a similarity of over 99%. Moreover, the sequence similarity with *Bacillus amyloliquefaciens* with accession number OR364403.1 reached 100.00%.The phylogenetic tree of 16S rDNA sequence of strain JL 54 was constructed (Fig. 9). The 16S rDNA of strain JL 54 and *Bacillus amyloliquefaciens* belonged to the same genetic branch.

#### Sequence analysis and phylogenetic tree construction of the *gyrB* gene

PCR amplification targeting the *gyrB* gene was performed using strain JL 54 as the template. The resulting amplification products were also sequenced by Sangon Biotech. Assembly of the sequencing data from the upstream and downstream primers was accomplished using DNA MAN software. The obtained target sequence had a length of 1,193 bp. The *gyrB* gene sequence information of strain JL 54 is available from NCBI under GenBank accession no. PP691101.

**Fig 10:**
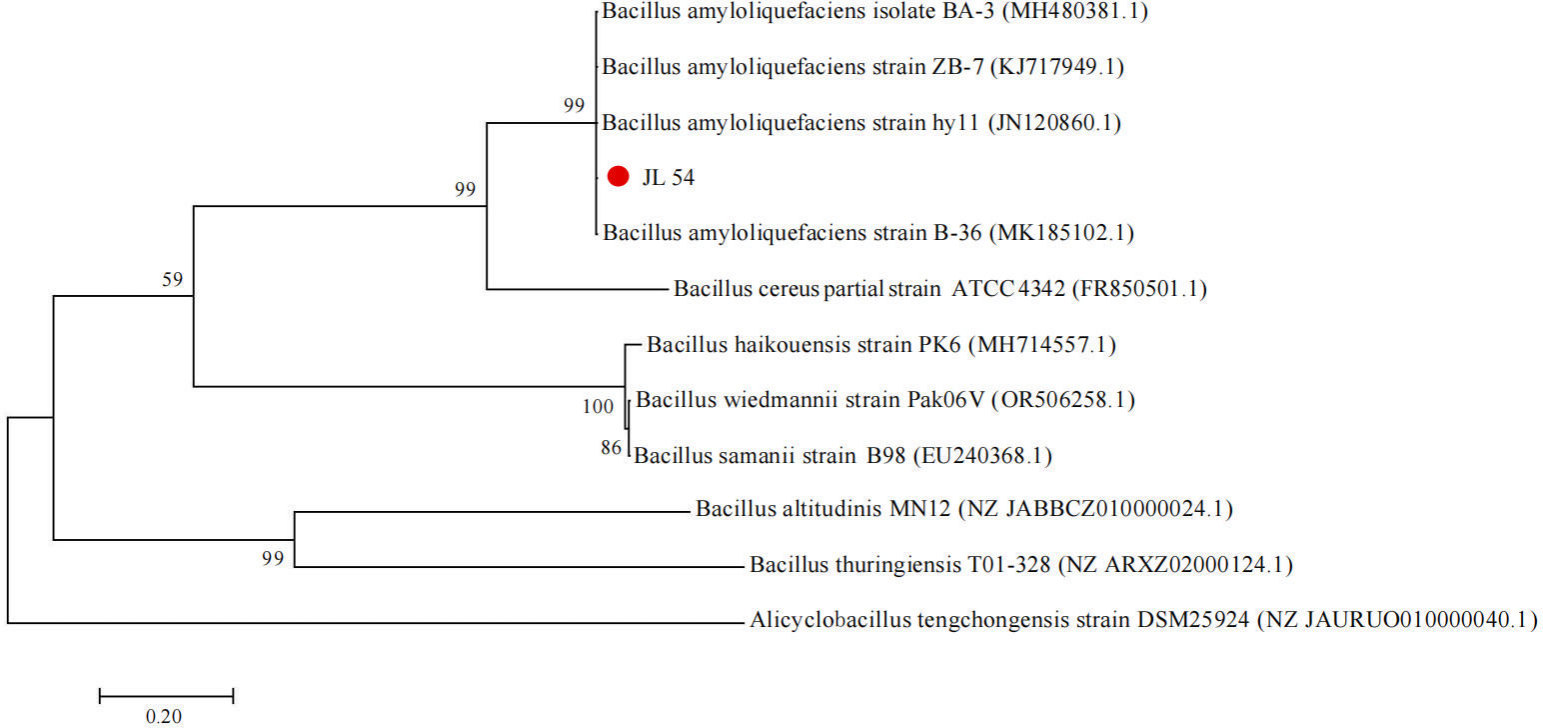
Phylogenetic tree of strain JL 54 based on BLAST results of the *gyrB* gene sequence.

We conducted a BLAST similarity alignment with known sequences on NCBI. The results showed that the *gyrB* gene sequence of strain JL 54 had the highest homology with *Bacillus amyloliquefaciens*, with a similarity of over 99%. Moreover, the sequence similarity with *Bacillus amyloliquefaciens* with accession number MH480381.1 reached 100.00%.The phylogenetic tree of *gyrB* sequence of strain JL 54 was constructed (Fig. 10). The *gyrB* sequence of strain JL 54 and *Bacillus amyloliquefaciens* belonged to the same genetic branch.

#### Phylogenetic tree construction by combining 16S rDNA and *gyrB* genes

**Fig 11:**
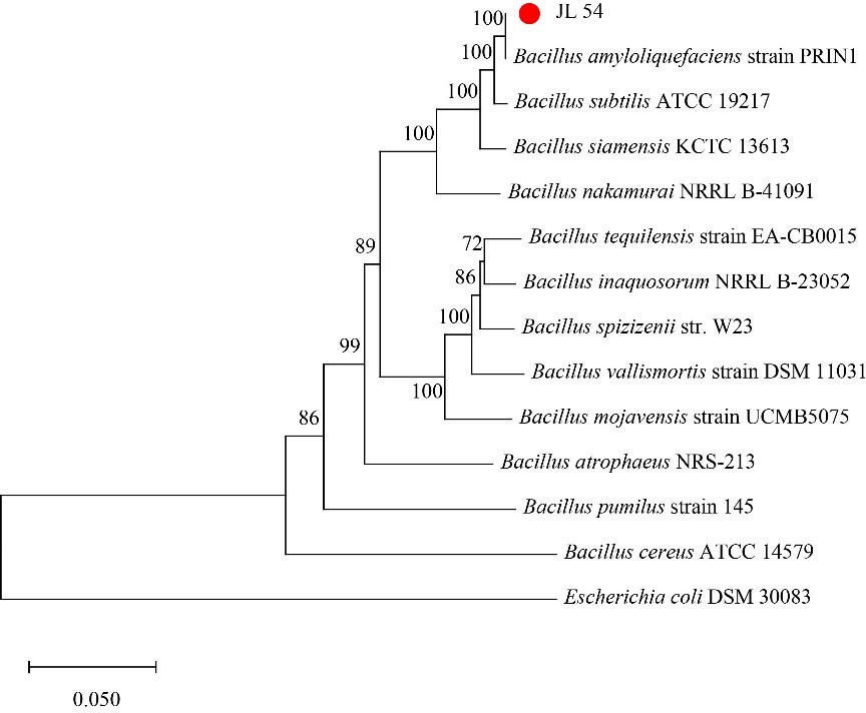
Phylogenetic tree of strain JL 54 based on the 16S rDNA-*gyrB* tandem feature sequence.

The 16S rDNA and *gyrB* gene sequences obtained above were connected to form a 16S rDNA-*gyrB* tandem feature sequence. A phylogenetic tree of strain JL 54 was constructed (Fig. 11). The two-gene tandem sequence of JL 54 strain also belonged to the same genetic branch as *Bacillus amyloliquefaciens*.

The results of colony characterization, 16S rDNA sequence, *gyrB* gene sequence, and 16S rDNA-*gyrB* tandem feature sequence analysis of the strain were combined. Strain JL 54 was identified as *Bacillus amyloliquefaciens*.

## DISCUSSION

For a long time, chemical control has been the main method in the prevention and control of plant diseases. The chemical germicides used in chemical control will cause serious pollution in the environment and at the same time will make the pathogens develop drug resistance. In recent years, biological control has been increasingly emphasized because of its environmentally friendly characteristics. Most of the isolation of biocontrol bacteria comes from the soil, but the research application of endophytic bacteria in plants is relatively few.

Larch shoot blight is a very important quarantine fungal disease in larch plantations in many countries around the world. It is extremely harmful and spreads rapidly. The control of larch shoot blight is still based on chemical control and forest management measures, and there is a lack of research on biological control of larch shoot blight. Liu et al. screened three fungi, *Sordaria filicola*, *Trichoderma atroviride*, and *Chaetomium globosum*, which are antagonistic to *Neofusicoccum laricinum* (19). Spraying 50% concentration of these three fungi suspension, the control effect was higher than 42.38%. In this experiment, an endophytic bacterium, *Bacillus amyloliquefaciens* JL 54, was isolated from larch. Moreover, the control effect of this bacterium on larch shoot blight in potted seedlings could reach 50%, which showed that this bacterium has a good prospect for biocontrol application.

*Bacillus* sp., with germicidal stability and compatibility with chemical germicides characteristics, is the main source of biological germicides and has been recognized as a very important microbial resource for biological control (27, 28). In the application of biocontrol, *Bacillus* spp. as a germicide showed better control effect. For example, the control effect of *Bacillus* sp. Bs-916 against rice sheath blight in the rice field was 73.9%–81.9% (29). In the application of endophytic bacteria in plant disease prevention and control, *Bacillus* spp. have also received more attention. In 2004, He et al. showed that the endophytic *Bacillus subtilis* BS-2 of chili peppers not only could colonize in cabbage but also had good growth-promoting and disease-prevention effects (30). In 2008, Chen et al. isolated a strain of *Bacillus subtilis* CQBS03 from citrus leaves, which had a good inhibitory effect on the growth and spores of *Xanthomonas axonopodis* pv. *Citri* (31). In 2019, Hu et al. isolated and screened an endophytic antagonistic bacterium from *Pinus tabulaeformis*, *Bacillus subtilis* (32). Li et al. isolated endophytic bacteria *Bacillus cereus* NJSZ-13 and *Bacillus pumilus* LYMC-3 with nematicidal activity on *Bursaphelenchus xylophilus* from pine stems (33, 34). The endophytic bacteria (*Bacillus altitudinis*) isolated from sugarcane by Nittaya et al. had a growth-promoting effect on sugarcane and an antagonistic activity against *Fusarium* (35). Dai et al. found that the secondary metabolites secreted by endophytic *Bacillus pumilus* HR 10 inhibited the growth and deformed the mycelium of *Sphaeropsis sapinea* and had a preventive effect on *Sphaeropsis* shoot blight of pine (36). In this experiment, *Bacillus amyloliquefaciens* was screened from 391 strains of larch endophytic bacteria as a significant antagonistic strain.

Studies have shown that *Bacillus amyloliquefaciens* can produce antimicrobial lipopeptide IturinA to inhibit the growth of *Rhizoctonia solani*, and can produce seven antimicrobial lipopeptides to inhibit the growth of *C. dematium*, *Xanthomonas campestris* pv. *campestris*, *Agrobacterium bumefaciens*, and *Pyricularia oryzae* (37, 38). Whether the antimicrobial substance of *Bacillus amyloliquefaciens* JL 54 obtained in this experiment against *Neofusicoccum laricinum* is an antimicrobial lipopeptide needs further study.

Combined with the results of prevention and safety test, strain NMG 23 was highly pathogenic to alfalfa and hemolytic, and was unsuitable for use as a biocontrol strain. Strains JL 6 and JL 54 had no pathogenicity to alfalfa and tobacco. Simultaneously, the two strains had no hemolysis. They were sensitive to 8 of the 10 antibiotics tested. Moreover, the control effect of JL 54 on larch shoot blight was significant in the experiment. Therefore, strain JL 6 is a safe strain, and strain JL 54 is a safe strain with great potential for biocontrol. The strain YN 2 showed incomplete hemolysis in the hemolysis test. Before the biocontrol strain will be put into field application, a large number of pathogenicity tests are needed to fully clarify its safety.

In this study, only the control effect of potted seedlings in a greenhouse was carried out for endophytic antagonistic bacteria. In the future, it is necessary to further study the control effect of those endophytic antagonistic bacteria in forests.

### Conclusion

In this study, 391 strains of endophytic bacteria were isolated from healthy larch branches and leaves from 13 sampling sites in 8 provinces of China. After preliminary and re-screening, 10 strains of endophytic bacteria were obtained; their inhibition rates against *Neofusicoccum laricinum* were all above 57%. Among them, strains YN 2, JL 6, NMG 23, and JL 54 exhibited the highest inhibition rates of 63.16%–65.08%. Therefore, these four strains were used as the research objects in the subsequent prevention and safety test.

In the greenhouse control effect experiment, it was found that strains YN 2 and JL 54 exhibited higher control effects on larch shoot blight after 14 days of inoculation with pathogenic fungi. The control effects of strains YN 2 and JL 54 were 57.7% and 50.0%, respectively. Combined with the results of control effect and safety test, strain JL 54 was considered to be a biologically safe bacterium and a promising strain for biocontrol. However, the virulence gene was detected in strain YN 2. A large number of pathogenicity tests are required to fully clarify the safety of this biocontrol strain before being put into the field application.

Because strain JL 54 is a potential and safe strain for biocontrol, it was selected for species identification in this experiment. Combined with the colony characteristics of the strain and the results of 16S rDNA sequence and *gyrB* gene sequence analysis, it was identified as *Bacillus amyloliquefaciens*.

## MATERIALS AND METHODS

### Biological materials

The tested larch seedlings were 3-year-old *Larix kaempferi* (Lamb.) Carrière, acquired in October 2022 from Longnan City, Gansu Province, China, and subsequently transplanted on into greenhouse pots on 15 October 2022. The non-flowering tobacco (*Nicotiana benthniciana*) and alfalfa (*Medicago sativa*) seeds were procured from Nanjing, Jiangsu Province, China.

The endophytic bacteria were isolated from branches and leaves of healthy larches collected from 13 sampling sites in 8 provinces of China (Yunnan, Hunan, Hebei, Jilin, Liaoning, Heilongjiang, Nei Mongol, and Shandong).

The pathogen of larch shoot blight is *Neofusicoccum laricinum* (Sawada) Y. Hattori & C. Nakash, with strain number DHKS 6-3. This strain was sourced from the Jilin Provincial Academy of Forestry Science and isolated from *Larix olgensis* Henry in Mudangang Forest Farm, Dunhua City, Jilin Province, China.

### Medium

1. Potato Dextrose Agar (PDA) medium: peeled potato 200 g, glucose 20 g, agar 20 g, distilled water 1 L.
2. NA medium: peptone 10 g, sodium chloride 5 g, beef extract 3 g, agar 20 g, pH 7.3, distilled water 1 L, pH 7.2–7.4.
3. LB medium: tryptone 10 g, yeast extract 5 g, sodium chloride 4 g, distilled water 1 L, pH 7.2–7.4.
4. Mueller-Hinton (MH) medium: beef powder 2 g, soluble starch 1.5 g, acid hydrolyzed casein 17.5 g, agar 20 g, distilled water 1 L, pH 7.2–7.4.
5. Blood agar medium: peptone 10 g, sodium chloride 5 g, beef extract 3 g, defibered sheep blood 50 mL, agar 20 g, distilled water 1 L, pH 7.2–7.4.

The growth and antagonism test of *Neofusicoccum laricinum* (Sawada) Y. Hattori & C. Nakash were conducted using PDA medium. The isolation and growth of endophytic bacteria were achieved using NA medium and LB medium, while the hemolysis test utilized blood agar medium. Additionally, MH medium was employed for drug sensitivity test (39).

### Isolation and purification of endophytic bacteria from larch

Endophytic bacteria were isolated by tissue separation method (40). The method started with selection of healthy larch branches and needles, then rinsing the surface of the larch plant tissue under running water to remove surface dirt. Subsequently, the leaves and branches were sterilized separately, peeling the outer skin of the branches for proper sterilization. Following this, the leaves and branches were washed sequentially with sterile water, disinfected with 75% ethanol for 60 s and 3% sodium hypochlorite for 30 s, and finally rinsed three times with sterile water. The sterile water obtained after the last washing should be used as a control and coated on NA medium. Next, the sterilized branches, cut into approximately 2-cm lengths, should be placed in NA medium, with a total of five branches positioned in the center and around each dish. Similarly, the leaves should be placed directly, homogenized, and coated. The disinfected leaves, also cut into approximately 2-cm lengths, should be placed on NA medium, with five leaves arranged in the center and around each dish. For homogenization, the leaf pulp was first ground with a sterilized mortar and pestle, and then 200 µL of leaf pulp was pipetted into the NA medium and spread uniformly with a spreader.

The treated NA medium was sealed and placed in a constant temperature incubator at 28℃ for incubation. After 2–3 days, the bacterial colonies that have grown in the medium were picked and incubated. Subsequently, after 1–2 days, the growth of the colonies was observed. A single colony was selected and inoculated on NA solid medium for purification. This purification process was repeated twice. Each endophytic bacterial solution was stored in an Eppendorf tube with 50% sterile glycerol and temporarily stored in a refrigerator at −20°C.

### Screening of antagonistic bacteria

The obtained strains underwent screening and re-screening using the plate confrontation method (41). *Neofusicoccum laricinum* (Sawada) Y. Hattori & C. Nakash was inoculated on PDA medium and cultured at 28°C for 5 days. Fungus cakes were prepared by punching the edge of the pathogenic fungal colony, the sterile puncher with a diameter of 6 mm.

To initiate the preliminary screening, the two-sided streaking confrontation culture method was employed. This involved inoculating the vigorously growing pathogen cake of larch shoot blight in the center of the PDA plate, while a single colony of endophytic bacteria was selected and streaked on both sides of the PDA plate. Additionally, a control plate without the streaking of endophytic bacteria strain, only inoculated with pathogenic fungi, was established. Each of the these procedures was repeated three times. Subsequently, the plates were incubated in a constant temperature incubator at 28℃. Upon reaching a 90-mm diameter in the control group’s petri dishes, we observed the formation of the inhibition zone in the treatment group and measured the diameter of the pathogenic fungal colonies in both the treatment and control groups to calculate the inhibition rate.

The rescreening process employed the four-point standoff culture method. In the center of a 90-mm diameter PDA plate containing 25 mL, the pathogen cake of *Neofusicoccum laricinum* was placed, and single colonies of endophytic bacteria were selected. The endophytic bacteria were then inoculated at four diagonals, positioned 3 cm away from the pathogen cake, with each treatment being repeated three times. Simultaneously, a plate with only pathogenic fungi cake placed in the center and no endophytic bacteria inoculation served as the control. Subsequently, the plates were incubated at 28℃ in a constant temperature incubator. The diameter of the pathogenic fungi colonies in both the treatment and control groups was measured, and the inhibition rate was calculated when the control group had reached a full petri dish length of 90 mm.

The colony diameters of the control and treated groups were measured using the cross method, and the inhibition rate was calculated using the following formula (42):


Inhibition rate=[(diameter of control colony−diameter of treatment colony) / (diameter of control colony−diameter of pathogen cake)]×100%

The inhibition rate of each endophytic bacteria was recorded. Subsequently, four strains of endophytic bacteria exhibiting the highest resistance to *Neofusicoccum laricinum* were selected for subsequent pot seedling control experiments.

### Pot experiment

The experiment was conducted in the greenhouse at Nanjing Forestry University from April to May 2023. Healthy larch seedlings with similar growth status were selected for pot and inoculation experiments. The experiment comprised four treatment groups and two control groups (one negative control group and one blank control group), each consisting of six plants.

Upon preservation at 4°C, the four strains of endophytic bacteria with the most potent antagonistic effect, as identified above in “Screening of antagonistic bacteria,” were inoculated on NA plates and placed in a constant temperature incubator at 28℃ to activate cultivation for 36 h. Following this, a ring of single colonies was selected and inoculated into conical flasks containing LB culture medium. The bacteria solution of the four endophytic bacteria strains was obtained by shaking the culture for 1 day at 28°C and 200 r/min. Subsequently, the bacteria solution of the four endophytic bacteria strains was withdrawn using a pipette gun and inoculated into sterilized and cooled LB culture medium at 1% inoculation amount. It was then placed on the shaker at 200 r/min and 28℃ under dark condition for 3 days, resulting in the fermentation broths of the four endophyte strains being obtained.

The antagonistic endophytic bacteria were applied using the root irrigation method, with the fermentation broth of antagonistic bacteria being pipetted into the root soil of pine seedlings five times. Conversely, the pathogenic fungus was inoculated using the wounding method (43). A tangential section was excised from the lower part of the branch using a sterile blade, and the pathogenic fungus cake was then affixed to the section, ensuring contact between the pathogenic fungus and the tangential section of the branch (Table 7). Subsequently, an inverted cone-shaped wrapping cake was fashioned using a sealing film, with distilled water added to facilitate moisturization and maintain the activity of the pathogenic fugus. The treated larch seedlings were then incubated at room temperature (28℃), while a humidifier was employed in the greenhouse to establish a high-temperature and high-humidity environment. Incidence was observed daily, and distilled water was added to the sealing film. After 14 days, the incidence rate, disease index, and control effect were calculated.

**TABLE 7:** Design of a trial program on the effectiveness of potted seedlings against treatment

Treatment	Experimental inoculation scheme
YN 2	Application of YN 2 fermentation liquid (1 × 10^7^ CFU/mL) 25 mL, inoculated with larch shoot blight fungus cake 2 days later
JL 6	Application of JL 6 fermentation liquid (1 × 10^7^ CFU/mL) 25 mL, inoculated with larch shoot blight fungus cake 2 days later
NMG 23	Application of NMG 23 fermentation liquid (1 × 10^7^ CFU/mL) 25 mL, inoculated with larch shoot blight fungus cake 2 days later
JL 54	Application of JL 54 fermentation liquid (1 × 10^7^ CFU/mL) 25 mL, inoculated with larch shoot blight fungus cake 2 days later
CK1	Application of sterile water 25 mL, inoculated with larch shoot blight fungus cake 2 days later
CK2	Application of sterile water 25 mL only

Larch shoot blight grading standard is as follows: Grade 0: whole plant disease-free; Grade 1 : less than 5% of the needles; Grade 2: 6%−25% needles; Grade 3: 26%−50% needles; Grade 4: 51%−75% of needles; Grade 5: more than 76% of the needles were infected.

The calculation formulas of incidence, disease index, and control effect are as follows (44):


Incidence rate =(number of diseased plants / total number of investigated plants) ×100%

Disease index=[∑(representative value of disease level×number of diseased plants at each level)]/(representative value of the highest level×total number of investigated plants)×100%

$$\begin{array}{l} Control\;effect=[(disease\;index\;of\;negative\;control\;group-disease\;index\;of\;treatment\;group)\;/\\ disease\;index\;of\;negative\;control\;group]\times 100\% \end{array}$$

### Safety detection of antagonistic bacteria

#### Tobacco inoculation

The activated antagonistic bacteria were inoculated into LB medium and cultured at 28°C with a rotation speed of 200 r/min for 24 h. Subsequently, the fermentation broth was centrifuged at 10,000 r/min for 10 min at 4°C to obtain a cell pellet. The concentration of cells was adjusted to 1 × 10^8^ CFU/mL using sterile water. Then, using a sterilized 1-mL syringe, 100-µL bacterial suspension was injected into the mesophyll cells from the lower epidermis of tobacco leaves. As a control, tobacco leaves inoculated with sterile water (CK) were utilized. The inoculated tobacco plants were incubated in an artificial climatic chamber set at 25° and 85% relative humidity and were subjected to a 16-h photoperiod. Three days following the inoculation, the tobacco leaves were inspected for the presence of spots.

#### Alfalfa inoculation

The selected alfalfa seeds underwent a 20-min soaking in 98% concentrated sulfuric acid. Subsequently, the seeds were thoroughly rinsed with a large volume of sterile water. The rinsed seeds were then placed in an appropriate amount of sterile water and subjected to incubation at 30°C with a rotation speed of 200 r/min for 8 h. Following this, the seeds were rinsed twice with sterile water and carefully transferred onto 1% water agar plates using sterile tweezers (45). Once the seeds sprouted, a small incision was made on the cotyledon using an inoculation needle. Then, 10 µL of prepared fermentation broths of YN 2, JL 6, NMG 23, JL 54, and LMG 1222, each with a concentration of 1 × 10^8^ CFU/mL, was applied to the wound. Each treatment group consisted of three replicates, with five seeds per replicate. The negative control comprised LB liquid medium, while the positive control used the fermentation broth of the control strain with pathogenic activity on alfalfa LMG 1222 (*Burkholderia cepacia*).

After the inoculation treatment, the alfalfa seeds were placed in an artificial climate incubator set at a temperature of 30°C and relative humidity of 90% and were subjected to timed light conditions (12 h of light and 12 h of darkness) for a duration of 10 days to monitor the growth of alfalfa.

#### Drug sensitivity test

The fermentation broth of YN 2, JL 6, NMG 23, and JL 54 strains, each at a concentration of 1 × 10^8^ CFU/mL, was evenly spread on the surface of MH agar plates using sterile cotton swabs. The plates were then placed on a sterile operating table and allowed to dry for 5 min. Antimicrobial drug sheets (Hangzhou Microbiology Reagent Co., Ltd.) were carefully retrieved using sterile tweezers and affixed onto the surface of the MH agar plates coated with the tested strains. After pasting, gently pressure was applied with tweezers to ensure the antimicrobial paper adhered securely. Each plate was equipped with five antimicrobial sheets, arranged in a uniform manner. The plates were subsequently incubated at 28°C. After 24 h, the presence or absence of bacteriostatic rings around the antimicrobial drug paper was observed, and the diameter of the bacteriostatic ring (including the diameter of the paper) was measured using a vernier caliper (46). Each treatment group consisted of three replicates, and the average diameter of the bacteriostatic ring was determined. The susceptibility of the strains to different antimicrobial drugs was assessed following the technical requirements outlined in the antimicrobial drug susceptibility test (Table 8).

**TABLE 8:** Judgment criteria for drug sensitivity testing^*a*^

Antibiotics	Paper drug content (μg/tablet)	Diameter of inhibition circle (mm)		
		R	I	S
Penicillin	10	<15	–^*b*^	≥15
Ampicillin	10	<14	14–16	>16
Cafamezin	30	<15	15–17	>17
Amikacin	30	<15	15–16	>16
Gentamicin	10	<13	13–14	>14
Erythromycin	15	<14	14–22	>22
Norfloxacin	10	<13	13–16	>16
Ciprofloxacin	5	<16	16–20	>20
Compound sulfamethoxazole	25	<11	11–16	>16
Chloroamphenicol	30	<13	13–17	>17

^*a*^ I, sensitive intermediary; R, resistant; S, sensitive.

^*b*^ "–" indicates no diameter of inhibition circle is classified in the sensitive intermediary.

#### Hemolysis test

The strains YN 2, JL 6, NMG 23, and JL 54 were inoculated onto blood agar plates and incubated in a constant temperature incubator at 28°C for 24 h. Subsequently, the plates were observed for the presence of hemolytic rings (47).

The hemolysis rate was determined by supernatant hemoglobin spectrophotometry (48).

Two milliliters of fresh blood was taken from sheep, anticoagulated with anticoagulant citrate dextrose solution (ACD), diluted with 2.5 mL of physiological saline, and refrigerated at 4℃. In the negative control group, 10 mL of normal saline (0.9% NaCl solution) was added to the test tube. In the positive control group, 10 mL of distilled water was added to the test tube. In the treatment group, 1 mL of the tested bacterial solution and 10 mL of normal saline were added to the test tube. All test tubes were placed in a constant temperature water bath at 37°C ± 1°C for 30 min. Diluted blood (0.2 mL) was added to each test tube, gently mixed, and placed in a water bath at 37.2°C ± 1°C for 1 h.

The liquid was poured out from the tube and centrifuged at 800 × *g* for 5 min. The supernatant was aspirated, and the absorbance at 545 nm was measured on a UV-Vis spectrophotometer, zeroed with physiological saline. The absorbance of the control group was taken as the average of three test tubes. The absorbance of the negative control should not exceed 0.03, and the absorbance of the positive control should be 0.8 ± 0.3; otherwise the experiment should be repeated.

The average absorbance of the negative control group is Dnc; the average absorbance of the positive control group is Dpc, and the absorbance of the sample is Dt. The formula for calculating the hemolysis rate (*Z*) is as follows:


Z=[(Dt−Dnc) / (Dpc−Dnc)]×100%

The hemolysis rate of the test sample should be less than 5%. A hemolysis rate of more than 5% indicates that the bacteria have hemolysis.

#### Detection of virulence gene

The presence of hemolytic enterotoxin genes (*hblA*, *hblB*, *hblC*, and *hblD*), non-hemolytic enterotoxin genes (*nheA*, *nheB*, and *nheC*), emetic toxin gene (*ces*), enterotoxin gene (*entFM*), and cytotoxin gene (*cytK*) was determined using PCR-specific amplification. The primer sequences used for PCR amplification of toxin genes are listed in Table 9. The amplified products were then analyzed by 1% agarose gel electrophoresis to confirm the presence of toxin genes in the antagonistic strains.

**TABLE 9:** Primers used for amplifying toxigenic genes

Target gene	Primer sequence (5′−3′)	Annealing temperature (°C)	Product length (bp)
*hblA*-F	GCAAAATCTATGAATGCCTA	53	884 (49)
*hblA*-R	GCATCTGTTCGTAATGTTTT		
*hblB*-F	AAGCAATGGAATACAATGGG	53	2,684 (50)
*hblB*-R	AATATGTCCCAGTACACCCG		
*hblC*-F	GATACTCAATGTGGCAACTGC	55	740 (50)
*hblC*-R	TTGAGACTGCTCGTCTAGTTG		
*hblD*-F	AATCAAGAGCTGTCACGAAT	55	430 (51)
*hblD*-R	CACCAATTGACCATGCTAAT		
*nheA*-F	TACGCTAAGGAGGGGCA	53	500 (51)
*nheA*-R	GTTTTTATTGCTTCATCGGCT		
*nheB*-F	CAAGCTCCAGTTCATGCGG	55	935 (51)
*nheB*-R	GATCCCATTGTGTACCATTG		
*nheC*-F	ACATCCTTTTGCAGCAGAAC	55	618 (49)
*nheC*-R	CCACCAGCAATGACCATATC		
*ces*-F	TTGTTGGAATTGTCGCAGAG	58	405 (52)
*ces*-R	GTAAGCGAACCTGTCTGTAACAACA		
*entFM*-F	ATTGCAGGTTTAGCAGCAGCTT	58	85 (53)
*entFM*-R	GCGCTTCATTTGAAACTTGTGC		
*cytK*-F	GCGCTGATAAACAGATTGCCGT	53	105 (53)
*cytK*-R	TAGCGCCAGGGATTGGGTAGTT		

### Morphological identification of superior antagonistic bacteria

The endophytic strains exhibiting effective control and high safety, as identified in section 2.6, were inoculated onto NA medium and incubated at 28°C for 48 h. The morphological characteristics of the colonies were observed and described, including size, shape, edge morphology, color, transparency, and surface properties. Gram staining was performed on the bacterial culture. Smears of single colony grown on NA plates for 24 h were fixed and subjected to staining procedures. A drop of crystal violet dye was applied for 1 min. Decolorization was achieved using 95% ethanol decolorization for 20–30 s, followed by washing with water. Subsequently, safranine re-dyeing agent was applied for 3 min, followed by washing, air-drying, and observation under oil immersion microscope.

### Molecular biology identification

#### Extraction of genomic DNA from endophytic bacterium

Genomic DNA of endophytic bacterium was extracted using Ezup Column Bacteria Genomic DNA Purification Kit (Sangon Biotech). The specific steps refer to the operation instructions.

The 16 S rDNA fragment was amplified and the bacterial universal primers 27 F (5′-AGAGAGTTTGATCCTGGCTCAG-3′) and 1492 R (5′-GGTTACCTTGTTACGACTT-3′) were selected. PCR amplification of the *gyrB* gene sequence was performed using primers UP-1 (5′ -GAAGTCATCATGACCGTTCTGCAYGCNGGNGGNAARTTYGA-3′) and UP-2r (5′-AGCAGGATACGGATGTGCGAGCCRTCNACRTCNGCRTCNGTCNGTCNGTCAT-3′) (54).

PCR reaction system (20 µL): Green Taq Mix 10 µL, upstream and downstream primers 1 µL, template DNA 2 µL, dd H2O 6 µL.

PCR reaction procedure of 16 S rDNA: pre-denaturation at 95°C for 5 min, denaturation at 95°C for 30 s, annealing at 48°C for 30 s, extension at 72°C for 90 s, 30 cycles, final extension at 72°C for 10 min.

PCR reaction procedure of *gyrB* gene sequence: pre-denaturation at 94°C for 5 min, denaturation at 94°C for 30 s, annealing at 60°C for 30 s, extension at 72°C for 90 s, 30 cycles, final extension at 72°C for 5 min.

#### Sequencing and phylogenetic analysis

The PCR-amplified fragments were sent to Sangon Biotech for sequencing. The 16S rDNA and *gyrB* gene sequences obtained by sequencing were subjected to BLAST alignment on NCBI. The 16S rDNA and *gyrB* gene sequences of representative strains were selected for multi-sequence homology comparison. The 16S rDNA and *gyrB* gene sequences obtained above were connected to form a 16S rDNA-*gyrB* tandem feature sequence (55). The phylogenetic tree was constructed using software MEGA, version 7, and the identification results of different phylogenetic evolutionary trees were compared (56).

### Data analysis

The results of the statistics were summarized and calculated using Excel software, version 2016, and one-way analysis of variance was performed using IBM SPSS Statistics (version 26; IBM Corp., Armonk, NY). The data were presented as the mean average of three replicates ± standard deviations. Data with the same letters mean the differences are not significant in the 5% level (Duncan multiple range test).
